# Prediabetes and diabetes mellitus type II after ischemic stroke

**DOI:** 10.1177/23969873241304301

**Published:** 2025-01-07

**Authors:** Kurt Moelgg, Anel Karisik, Lukas Scherer, Lucie Buergi, Benjamin Dejakum, Silvia Komarek, Julian Granna, Christian Boehme, Raimund Pechlaner, Thomas Toell, Michael Knoflach, Stefan Kiechl, Susanne Kaser, Alexander Egger, Andrea Griesmacher, Lukas Mayer-Suess

**Affiliations:** 1 Department of Neurology, Medical University of Innsbruck, Innsbruck, Austria; 2 VASCage, Centre on Clinical Stroke Research, Innsbruck, Austria; 3 Department of Internal Medicine I, Medical University of Innsbruck, Innsbruck, Austria; 4 Central Institute of Clinical Chemistry and Laboratory Medicine, Medical University of Innsbruck, Innsbruck, Austria

**Keywords:** Prediabetes, type II diabetes mellitus, cardiovascular risk factors, ischemic stroke, secondary prevention, antidiabetic therapy

## Abstract

**Introduction:**

The progression of diabetes status in post-stroke patients remains under-investigated, particularly regarding new treatments for type II diabetes mellitus (DM II), like glucagon-like peptide 1 receptor agonists (GLP-1-RA) and sodium-glucose co-transporter-2 (SGLT-2) inhibitors, which have not been studied in the post-stroke setting.

**Patients and methods:**

Eight hundred eighty-four consecutive ischemic stroke patients recruited to our prospective STROKE-CARD Registry were assessed concerning their glycemic status at baseline (normoglycemia, prediabetes, DM II) and change over time within 1 year follow-up. Multivariate logistic regression was performed to identify factors associated with transitioning from normoglycemia to prediabetes or DM II. Additionally, we reviewed ongoing clinical trials for GLP-1-RA and SGLT-2 inhibitors in the context of acute ischemic stroke.

**Results:**

At baseline, 44.6% (*n* = 394) of individuals had normoglycemia, 33.9% (*n* = 300) were prediabetic, and 21.5% had DM II (*n* = 190). After 1 year, normoglycemia decreased by 12.1 percentage points (*n* = 107), whereas prediabetes and DM II increased by 10.2 percentage (*n* = 90) points and 1.9 percentage points (*n* = 17), respectively. Statin therapy was the only significant risk factor for progression. 23.4% (*n* = 207) of our cohort would have met eligibility criteria for a recent trial on semaglutide in obese non-diabetics with prior cardiovascular disease. However, only one ongoing trial aims at evaluating short-term cardiovascular risk reduction in stroke patients.

**Discussion:**

GPrediabetes and DM II are frequent in ischemic stroke patients. Even within an intensified post-stroke disease management setting, a considerable amount of stroke survivors convert to prediabetes or DM II within the first year. Our results demonstrate a notable proportion of patients qualifying inclusion in studies examining the efficacy of GLP-1-RA agonists and SGLT-2 inhibitors in secondary prevention.

**Conclusion:**

Given the high prevalence and progression of prediabetes and DM II in stroke survivors, there is a need for clinical trials evaluating the use of GLP-1-RA and SGLT-2 inhibitors in this population.

## Graphical abstract

**Graphical abstract:**
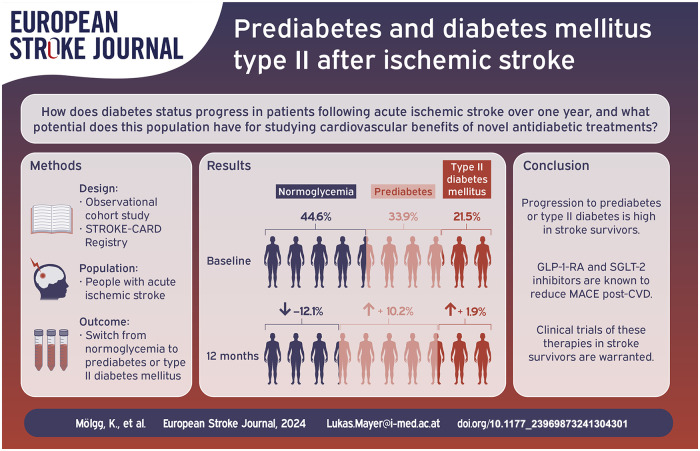

## Introduction

Ischemic stroke represents one of the foremost contributors to global mortality, morbidity and entails a significant economic burden.^1,2^ A large meta-analysis, incorporating >350,000 individuals reported that 33% of stroke patients are diabetic at admission.^3^ Over the last decade, treatment for DM II has been reshaped with glucagon-like peptide 1 receptor agonists (GLP-1-RA) and sodium-glucose co-transporter-2 inhibitors (SGLT-2 inhibitors) emerging as beneficial in reducing the risk of incident cardiovascular disease (CVD) in patients with known type II diabetes or, in case of the GLP-1-RA semaglutide in non-diabetic individuals with prior CVD and high BMI (⩾27 kg/m^2^).^4–12^ These investigations prompt calls for the development of customized interventions applying such drugs to post-CVD setting independent of glycemic status. However, little is known about the longitudinal dynamics of prediabetes and type DM II within 1 year after acute ischemic stroke. Hence, our aim was to provide such evidence and characterize the number of potentially eligible individuals meeting the inclusion criteria for a post-stroke centered efficacy trial in the STROKE-CARD Registry, a prospective multi-center cohort of consecutive ischemic stroke survivors.

## Patients and methods

### Study population

The STROKE-CARD Registry (ClinicalTrials.gov ID NCT04582825) is a prospective observational study initiated in December 2020 at the Medical University of Innsbruck and Hospital St. John’s of God Vienna.^13^ It includes consecutive patients with ischemic stroke or high-risk transient ischemic attack (TIA) (ABCD^2^-score ⩾ 4) within 30 days prior of admission to participating centers. Written informed consent is obtained from all participants. Its protocol is based on the STROKE-CARD trial, which established a structured post-stroke disease management program.^13^ It includes comprehensive clinical and laboratory work-up during the initial hospital stay, followed by in-person follow-ups at 3- and 12-months after the index event, enabling individualized post-stroke treatment and personalized risk factor management through stroke experts, physicians, and rehabilitation specialists.

The STROKE-CARD Registry has been approved by the Ethics Committee of the Medical University of Innsbruck (EK-Nr: 1182/2020) and complies with the Declaration of Helsinki.

### Variable definitions

We included acute ischemic stroke patients with completed 12-month follow-up recorded within the STROKE-CARD Registry at the Medical University of Innsbruck. The assessment of known pre-existing conditions (i.e. atrial fibrillation, arterial hypertension, dyslipidaemia, diabetes mellitus) as well as smoking status are based on detailed patient interview, medical history, and evaluation of prior medication. Prior and concomitant medication prescriptions were assessed based on inpatient- or telephone interview as well as review of electronic health records. Prediabetes was defined as hemoglobin A1c (HbA1c) values of 5.7% to 6.4% and DM II as levels ⩾6.5% at baseline. The guideline conform definition of DM II through blood glucose levels was not considered, as fasting plasma glucose levels may be elevated after acute ischemic stroke due to activation of the hypothalamic-pituitary-adrenal axis.^14–16^ Obesity was defined on BMI according to WHO cut-offs as follows: underweight (BMI < 18.5 kg/m^2^), normal weight (BMI = 18.5–24.9 kg/m^2^), pre-obese (BMI = 25–29.9 kg/m^2^), obese (BMI ⩾ 30 kg/m^2^).^17^ Arterial hypertension was defined according to guidelines as a blood pressure above ⩾140/90 mmHg.^18^ Dyslipidaemia at baseline was defined according to the following criteria: total cholesterol ⩾ 200 mg/dl, LDL-C ⩾ 100 mg/dl, HDL-C ⩽ 40 mg/dl, TG ⩾ 150 mg/dl, or Lp(a) ⩾ 75 nmol/l. Laboratory measurements were done at baseline, 3-month, and 12-month follow-up visit using standard laboratory methods, which include but are not limited to features reportedly being associated with developing DM II (i.e. total cholesterol, LDL-C, HDL-C, Lp(a), TG, vitamin D, erythrocyte sedimentation rate, CRP, leukocytes, bilirubin).^19–23^

Concerning study outcomes during follow-up, an intra-individual assessment of laboratory measures at 3- and 12-months post-stroke was used to identify longitudinal dynamics of glycaemic state. Definitions of prediabetes mirrored those of baseline assessments, with an addition single fasting plasma glucose level of 100 mg/dl–126 mg/dl at this time-point being considered as prediabetic as well.^15^ DM II during follow-up was defined according to current guidelines as HbA1c ⩾6.5% on two different days, fasting plasma glucose ⩾126 mg/dl on two different days, HbA1c ⩾6.5% and fasting plasma glucose above 126 mg/dl on a single day, or clinical diagnosis made by a physician during the follow-up period.^15,16^

### Measurement of HbA1c and fasting plasma glucose

HbA1c was measured in fasting patients using ethylenediaminetetraacetic acid blood samples and reported as percentage (%) aligned to the assay used in the Diabetes Control and Complications Trial. High-performance liquid chromatography at the Medical University of Innsbruck’s central laboratory was used, where HbA1c fractions were separated by porous cation-exchange column and detected via ultraviolet/visible detector.

Fasting plasma glucose levels were measured in lithium-heparin plasma samples using an enzymatic reaction method, where hexokinase phosphorylates glucose to produce glucose-6-phosphate, which is subsequently oxidized to gluconate-6-phosphate by glucose-6-phosphate dehydrogenase, generating NADPH. The NADPH formation rate, proportional to glucose concentration, was measured photometrically. All laboratory analysis were completed on the same day as sample collection to ensure optimal accuracy and reliability of the results.

### ClinicalTrial.gov search strategy

To identify ongoing and future clinical trials investigating efficacy of GLP-1-RA or SGLT-2 inhibitors in ischemic stroke, a targeted search was conducted on ClinicalTrials.gov using the following search terms:

- Ischemic Stroke & GLP-1-RA
- Ischemic Stroke & Glucagon-like peptide 1 receptor agonists
- Ischemic Stroke & Semaglutide/Liraglutide/Exenatide/Dulaglutide/Lixisentide
- Ischemic Stroke & SGLT-2 inhibitor
- Ischemic Stroke & Sodium-glucose cotransporter 2 inhibitor
- Ischemic Stroke & Empaglifozin/Dapaglifozin/Canaglifozin/Sotaglifozin/Ertuglifozin

### Statistical analysis

Age, BMI, Waist-to-Hip ratio (WtH) were analyzed by presenting descriptive statistics such as median and interquartile range (IQR). Categorical variables, including sex, pre-existing risk factors, and newly diagnosed risk factors were summarized using frequencies and percentages.

The prevalence of normoglycaemia, prediabetes, and DM II at baseline or follow-up were calculated as absolute values and proportions. Subgroup analysis differentiated between first-ever ischemic stroke patients and those with prior ischemic stroke.

Multivariate logistic regression analysis identified factors associated with the transition from normoglycemia to prediabetes or DM II over 12-months. The included variables were selected a priori based on literature-established associations with glycaemic changes: age, BMI, WtH, HDL cholesterol, leukocyte count, TG, smoking status, elevated fatty liver index (FLI), family history of diabetes, sex, and intake of beta-blockers or statins.

Variance Inflation Factor (VIF) was calculated for all covariates included to assess multicollinearity among predictors using a threshold of 3 to identify any significant collinearity concerns. For missing values in laboratory measurements, we applied the last value carried forward (LVCF) method, given the relative stability of these variables over the follow-up period. For BMI, we used mean imputation to retain the overall sample size and minimize data loss.

All calculations were performed using R (version 4.3.3, The R Foundation for Statistical Computing, Vienna, Austria).

## Results

A total of 884 individuals with acute ischemic stroke and complete 12-month follow-up within the STROKE CARD Registry were available. Approximately 96.4% of included patients attended the 3-month visit and 86.5% attended the 12-month visit (see Online Supplement – Supplementary Figure 1). Table 1 presents demographics, risk factors, and key patient characteristics of the cohort, including pre-defined subgroups.

**Table 1.** Baseline characteristics of study population.

Characteristics	All	First ever ischemic stroke	Prior ischemic stroke
	*N* = 884, %, median (IQR)	*N* = 697, %, median (IQR)	*N* = 187, %, median (IQR)
Age (years)	73 (62, 80)	72 (61, 80)	74 (65, 80)
Sex			
Men	570 (64.5)	436 (62.6)	134 (71.7)
Women	314 (35.5)	261 (37.4)	53 (28.3)
Body composition			
Body-Mass-Index (kg/m^2^)	25.67 (23.53, 28.43)	25.49 (23.49, 28.39)	25.96 (23.94, 28.6)
Waist-to-hip ratio	0.94 (0.88, 0.99)	0.94 (0.88, 0.99)	0.95 (0.89, 0.99)
Trial of Org 10172 in Acute Stroke Treatment (TOAST) classification			
Large-artery atherosclerosis	175 (19.8%)	132 (18.9%)	43 (23.0%)
Cardioembolism	187 (21.2%)	147 (21.1%)	40 (21.4%)
Small-artery occlusion	177 (20.0%)	146 (20.9%)	31 (16.6%)
Stroke of other determined etiology	45 (5.1%)	36 (5.2%)	9 (4.8%)
Stroke of undetermined etiology	300 (33.9%)	236 (33.9%)	64 (34.2%)
Pre-existing risk factors			
DM II	152 (17.1%)	99 (14.2%)	53 (28.3%)
Hypercholesterinaemia	430 (48.6%)	287 (41.2%)	143 (76.5%)
Atrial hypertension	733 (82.9%)	578 (82.9%)	155 (82.9%)
Smoking	188 (21.3%)	149 (21.6%)	39 (20.8%)
Atrial fibrillation	107 (12.1%)	78 (11.2%)	29 (15.5%)
Newly diagnosed conditions during in-hospital stay			
DM II	38 (4.3)	36 (5.2)	2 (1.1)
Hypercholesterinaemia	380 (43.0)	347 (49.8)	33 (17.6)
Atrial hypertension	104 (11.8)	91 (13.1)	13 (7.0)
Atrial fibrillation	59 (6.7)	50 (7.2)	9 (4.8)
Family history of DM II	165 (18.7)	128 (18.4)	37 (19.8)

DM II: diabetes mellitus type II; IQR: interquartile range.

**Figure 1.**
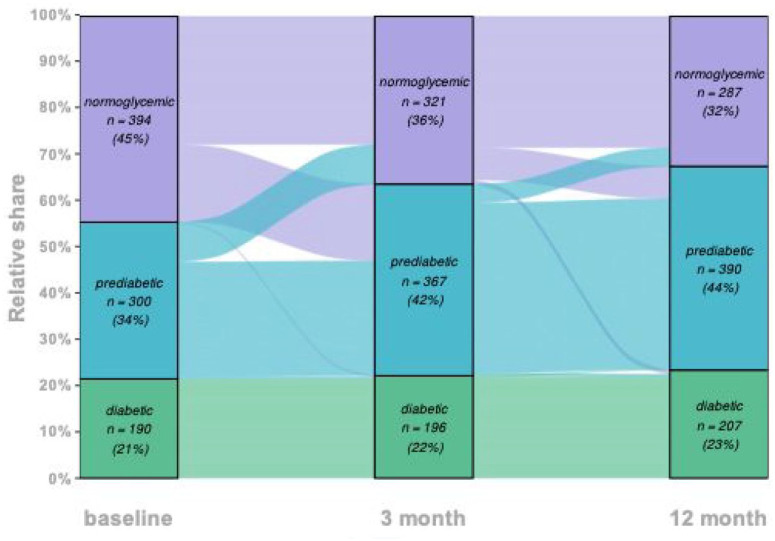
Longitudinal evolution of normoglycemic, prediabetic and diabetics individuals.

At baseline, 44.6% (*n* = 394) were normoglycemic, 33.9% (*n* = 300) had prediabetes, and 21.5% (*n* = 190) had DM II, of which 17.2% (*n* = 152) were known prior to stroke and 4.3% (*n* = 38) were novel diagnoses. Figure 1 illustrates the shift in these values from baseline to 3- and 12-month follow-up. Over 1 year, DM II increased by 1.9 percentage points (pp, 95% CI [−2.0–5.8], *n* = 17). However, a notable rise in prediabetic patients (10.2 pp, 95% CI [5.7–14.7], *n* = 90) along with a significant decrease in normoglycemic individuals was seen (12.1 pp, 95% [CI 7.6–16.6], *n* = 107).

After discharge, intake of high-potency statins was nearly twice as high as at admission, and this tendency remained stable over the 12-month period (see Online Supplement – Supplementary Figure 2). Similarly, oral antidiabetic therapy increased after discharge compared to admission and remained stable over the following year (see Online Supplement – Supplementary Figures 3 and 4).

In multivariate logistic regression (Table 2), only statin intake was significantly associated with transitioning from normoglycemia to prediabetes or DM II (odds ratio [OR] 2.51, 95% CI – 1.08–6.38, *p* = 0.040). No statistically significant associations were found between the incidence of prediabetes or DM II and literature-established risk factors for developing type DM II (Table 2).^19,20,24–26^

**Table 2.** Multivariate logistic regression of individuals transitioning from normoglycemia to prediabetes or type II diabetes mellitus.

Characteristic	First ever ischemic stroke		All stroke patients	
	OR (95% CI)^a^	*p*-Value	OR (95% CI)^a^	*p*-Value
Body-Mass-Index	1.01 (0.92–1.11)	0.78	1.01 (0.93–1.09)	0.87
Age	1.02 (1.00–1.04)	0.064	1.01 (0.99–1.03)	0.23
HDL-C	0.98 (0.96–1.00)	0.064	0.99 (0.97–1.00)	0.16
Leukocytes	1.07 (0.94–1.22)	0.33	1.11 (0.99–1.25)	0.066
Triglycerides	1.00 (0.99–1.00)	0.47	1.00 (0.99–1.00)	0.78
Waist-to-hip ratio	1.69 (0.04–81.5)	0.79	7.28 (0.22–276)	0.27
Smoker	1.00 (0.52–1.92)	0.99	0.92 (0.51–1.64)	0.77
Beta-blockers	0.82 (0.47–1.43)	0.49	0.96 (0.59–1.55)	0.86
Statins	2.69 (1.02–8.00)	0.056	2.51 (1.08–6.38)	0.040
Elevated Fatty Liver Index	1.14 (0.57–2.26)	0.72	1.05 (0.56–1.96)	0.89
Family history diabetes	1.60 (0.84–3.09)	0.16	1.60 (0.89–2.90)	0.12
Sex				
Male	–		–	
Female	1.34 (0.73–2.47)	0.35	1.22 (0.70–2.13)	0.49
Trial of Org 10172 in Acute Stroke Treatment (TOAST) classification				
Large-artery atherosclerosis	–		–	
Cardioembolism	1.06 (0.48–2.31)	0.89	1.15 (0.58–2.31)	0.69
Small-artery occlusion	0.68 (0.32–1.43)	0.31	0.90 (0.45–1.80)	0.77
Stroke of other determined etiology	0.74 (0.24–2.25)	0.60	0.83 (0.30–2.20)	0.70
Stroke of undetermined etiology	0.81 (0.41–1.63)	0.56	0.82 (0.43–1.55)	0.54

Level of significance for these analyses was *p*<0.05.

### Clinical trials of GLP-1-RA or SGLT-2 inhibitors in post-stroke setting

Our ClinicalTrials.gov based review of ongoing studies revealed six studies investigating the effects of GLP-1-RA and SGLT-2 inhibitors with DM II in the post-stroke setting (see Online Supplement Supplementary Table 1). However, only one study is examining the efficacy of liraglutide in reducing CVD outcomes.

**Figure 2.**
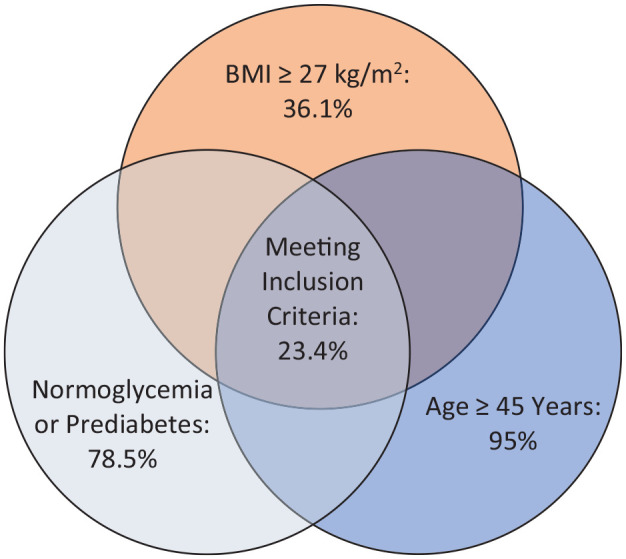
Venn diagram for fulfillment of inclusion criteria.

Currently, there are no planned studies evaluating the efficacy of GLP-1-RA and SGLT-2 inhibitors in a post-stroke secondary prevention setting. When considering eligibility of post-stroke patients in studies assessing safety and efficacy of GLP-1-RA and SGLT-2 inhibitors, a clinical trial would be able to recruit roughly a quarter of our study population (207 of 884, 23.4%), if inclusion criteria would mimic those of a recent study on the efficacy of semaglutide on CVD outcome in obese adults above the age of ⩾45 years without DM II (i.e. BMI ⩾ 27 kg/m² and normoglycemia or prediabetes at baseline) (Figure 2).^12^

## Discussion

In 884 consecutive ischemic stroke patients 17.2% (*n* = 152) had DM II and 33.9% (*n* = 300) had prediabetes at stroke onset. The only significant risk factor for transitioning from normoglycemia to prediabetes or type II diabetes mellitus was statin intake. This risk has been previously described in individuals undergoing low-intensity or high-intensity statin therapy which have their glycemic indices near the relative diagnostic threshold for PD or DM II, without affecting the cardiovascular benefit of statin therapy.^27^

Our DM II prevalence aligns with two Austrian studies (e.g. STROKE CARD trial)^13,28^ but is lower than the prevalence of 33% (CI 28%–38%) in a meta-analysis of 17 studies.^3^ However, our cohort suggests that one in three stroke patients is prediabetic in the acute phase, which harbours significance. Ranges of 31% to 53% in the acute phase after TIA or ischemic stroke have previously been reported. Still, varying definitions of prediabetes in these reports make comparisons or validation efforts difficult.^29,30^ Nevertheless, a nearly two-increased risk of recurrent stroke in patients with TIA or minor ischemic stroke was seen if non-fasting random plasma glucose levels indicative of impaired glucose tolerance were evident. This increased to threefold if levels were in DM II ranges.^31^ This is alarming as our study emphasizes that a considerable number of patients shift away from normoglycemia over time post-stroke (Figure 1).

Adequate post-stroke treatment through lifestyle and pharmacological intervention may limit the progression to prediabetes and DM II, reducing long-term stroke recurrence risk. GLP-1-RA or SGLT-2 inhibitors have positively impacted vascular outcomes in patients with DM II and CVD, warranting investigation in post-stroke cohorts.^12^ Currently, we identified no planned phase III studies for GLP-1-RA or SGLT-2 inhibitors in this setting. This is of note as our second key finding reveals that, when using the identical inclusion criteria applied in efficacy trials for semaglutide in preventing cardiovascular events, one out of four patients in our study population would be eligible for recruitment. This clearly underlines the feasibility of such trials in the post-stroke setting (Figure 2).^12^ Considering the conversion rate over time and the setting in which our analysis took place (i.e. within a post-stroke disease management program encapsulating post-stroke risk factor management) the number of potentially eligible patients over time may even be underestimated.

As previously stated, only one trial investigates the effect of GLP-1-RA antagonists on stroke recurrence. Though valuable, a trial with extended follow-up in a stroke specific cohort would be fruitful as has been shown in other all-cause CVD cohorts. This would harbour the opportunity to also address specific secondary post-stroke outcomes such as the effect of these novel treatments on, for example, cognitive deterioration and recurrent stroke risk. Coherently, our study indicates feasibility of such future trials in specific settings where in-depth post-stroke disease management measures are in place for all-cause stroke patients, which was not entirely the case in other prior studies investigating similar hypotheses. The strength of our study lies in its inclusion of consecutive ischemic stroke patients. Additionally, our study benefits from the standardized in-person follow-up of patients in a specialized out-patient clinic with clear standard operation procedures, allowing for an accurate longitudinal assessment of outcomes. Moreover, the diagnosis of DM II was based on laboratory parameters in accordance with current international guidelines, while prior studies relied solely on a single HbA1c value above 6.5%. This further enhances the reliability and clinical relevance of our findings.^3,32^ Concerning limitations, the definition of prediabetes based on a single HbA1c, or fasting plasma glucose value is limited by the regression toward the mean. This limitation was partially addressed by measurements of HbA1c and fasting plasma glucose levels at different time points. Limitations include the use of BMI as the sole criterion for defining obesity, as it been shown to leads to misclassification of individuals body constitution.^33^

Despite the limitations, our analysis provides valuable insights into the prevalence of DM II and prediabetes in acute ischemic stroke patients as well as its longitudinal evolution within the first-year post-stroke. Further, it implies that future studies investigating pharmacological intervention (i.e. through for instance GLP-1-RA or SGLT-2 inhibitors) in the post-stroke setting are feasible.

## Conclusion

Our study emphasizes that prediabetes as well as DM II are frequent in a large proportion of ischemic stroke patients. Even within an intensified post-stroke disease management setting, a considerable amount of post-stroke patients converts to prediabetes or DM II within the first year. Our results demonstrate a notable proportion of patients qualifying for a potential inclusion in studies examining the efficacy of GLP-1-RA agonists and SGLT-2 inhibitors in secondary prevention of cardiovascular events for ischemic stroke patients, indicating feasibility of future clinical trials.

## Supplemental Material

sj-docx-1-eso-10.1177_23969873241304301 – Supplemental material for Prediabetes and diabetes mellitus type II after ischemic stroke Supplemental material, sj-docx-1-eso-10.1177_23969873241304301 for Prediabetes and diabetes mellitus type II after ischemic stroke by Kurt Moelgg, Anel Karisik, Lukas Scherer, Lucie Buergi, Benjamin Dejakum, Silvia Komarek, Julian Granna, Christian Boehme, Raimund Pechlaner, Thomas Toell, Michael Knoflach, Stefan Kiechl, Susanne Kaser, Alexander Egger, Andrea Griesmacher and Lukas Mayer-Suess in European Stroke Journal
