# Supplementary material for: Prediabetes and diabetes mellitus type II after ischemic stroke
Source: Eur Stroke J. 2025 Jan 7;10(3):822–8. doi: 10.1177/23969873241304301 (PMC11705302; doi:10.1177/23969873241304301)
Supplement: sj-docx-1-eso-10.1177_23969873241304301 – Supplemental material for Prediabetes and diabetes mellitus type II after ischemic stroke [file sj-docx-1-eso-10.1177_23969873241304301.docx]

**Prediabetes and Diabetes Mellitus Type II after Ischemic Stroke**

**Online Supplement**

**Supplementary Figure 1. Patient Follow-Up Flowchart**

Patient included in the analysis

N = 884

Electronic healthcare data

N = 26

Telephone follow-up

N = 6

In-patient follow-up

N = 852

3-months follow-up

Electronic healthcare data

N = 78

Telephone visit

N = 41

In-patient follow-up

N = 765

12-months follow-up

**Supplementary Table 1. Identified Ongoing Clinical Trials with Investigating the Efficacy of GLP-1-RA or SGLT-2 inhibitors in Ischemic Stroke Patients.**

| **Study Name** | **NCT-Number** | **Inclusion Criteria of Identified Clinical Trials** | **Location** | **Setting (According to ClinicalTrials.gov)** | **Duration [Start Date (Actual) to (Estimated) Completion Date]** | **Current Trial Status on ClinicalTrials.gov** |
| --- | --- | --- | --- | --- | --- | --- |
| Liraglutide in Acute Minor Ischemic Stroke or High-risk Transient Ischemic Attack Patients With Type 2 Diabetes Mellitus (LAMP) | NCT03948347 | - Adult subjects (male or female ≥ 50 years); - Acute ischemic stroke patients (NIHSS ≤ 3 at the time of randomization) with type 2 diabetes mellitus within 24 hours of symptoms onset; - High-risk TIA patients (ABCD2 score ≥ 4 at the time of randomization) with type 2 diabetes mellitus within 24 hours of onset; - First stroke, or prior stroke without sequel (mRS score ≤ 1) and does not affect the NIHSS score; - Informed consent signed. | - Guangzhou, Guangdong, China | Prospective Multicentre Randomized Blank Controlled, Blinded Endpoint Study | 25.06.2019 to dd.06.2024 | Recruiting |
| Acute Subcutaneous SemaglutidE in Acute Ischemic sTroke (ASSET) | NCT05630586 | - Male and female patients (≥ 18 years) at the time of signed informed consent/proxy consent - Acute ischemic stroke with disabling neurological deficits (defined as an impairment of one or more of the following: language, motor function, cognition, gaze, vision, neglect, or ataxia) - Onset/last seen well to randomization < 4.5 hours - None to moderate disability in daily living before symptom onset (pre-stroke modified Rankin Scale 0-3) | - Aarhus University Hospital, Denmark - Bispebjerg Hospital, Denmark - Glostrup University Hospital, Denmark - Odense University Hospital, Denmark - Herning Hospital, Denmark - Aalborg University Hospital, Denmark - Rigshospitalet, Denmark | A Multicentre, Phase 2, Prospective, Randomized, Open-label, Blinded Endpoint Trial | 12.04.2022 to dd.12.2027 | Not Yet Recruiting |
| Glucagon-like Peptide 1 Receptor Agonist in Acute Large Vessel Occlusion Stroke Treated by Reperfusion Therapies (GALLOP) | NCT05920889 | - LVO stroke at terminal ICA or proximal M1 eligible for emergency endovascular treatment as per current treatment guideline. - LKW-to-puncture time ≤ 12 hours. - Age 18 years or greater. - National Institute of Health Stroke Scale (NIHSS) ≥10 - LVO stroke due to thromboembolism or intracranial stenosis (acute or acute on chronic occlusion). - Patients who received computer tomographic angiography and perfusion (CTA+P). - Pre-stroke (24 hours prior to stroke onset) independent functional status with modified Rankin Scale (mRS) ≤ 2. - Consent process completed as per national laws and regulation and the applicable ethics committee requirements. | - Chinese University of Hong Kong, Hong Kong - Linyi People's Hospital, China | Multicenter, Randomized Open-Label Pilot Study | 23.04.2023 to 31.12.2026 | Recruiting |
| The Effect of GLP-1 Receptor Agonist on Cerebral Blood Flow Velocity in Stroke (EGRABIS1) | NCT02829502 | - Patients ≥ 18 years with newly symptoms of stroke - Able to receive exenatide/placebo within 21 days after onset of symptoms - Radiological confirmed diagnoses of ischemic stroke - NIHSS between 1-20 at the onset of symptoms - modified rankin scale (mRS) ≤ 2 prior to onset of symptoms - Has given written informed consent | - Herlev-Gentofte Hospital, Denmark | Double-Blinded, Randomized, Controlled Pilot Trial | dd.08.2016 to dd.11.2023 | Recruiting |
| Pioglitazone and SGLT2 Inhibitors vs. DPP4 Inhibitors in Patients With Stroke | NCT04419337 | - - Ischemic stroke within 3 months of randomization   - Type 2 diabetes mellitus and Hba1C > 7.0% and under metformin therapy currently or previously   - Estimated glomerular filtration rate (eGFR) ≥ 45 mL/min/1.73 m2   - Age ≥ 20 y at study entry   - Ability and willingness to provide informed consent | - Chang Gung Memorial Hospital, Taiwan | Multicenter randomized, parallel-group study | 15.09.2019 to 31.01.2024 | Recruiting |
| Dapagliflozin Effect in Cognitive Impairment in Stroke Trial (DECIST) | NCT05565976 | - Ages of 60 and 80 years - Both sexes - Cerebrovascular ischemic event within 15 days - Clinical dementia rating score ≤ 0.5 - Signed informed consent | - Mondragon, Universidad de Guanajuato, Mexico | Single Center, Double-blind, Randomized, placebo-controlled, parallel-group. | 01.08.2020 to 01.07.2025 | Recruiting |

**Supplementary Figure 2. Statin Therapy in All Patients**

**Supplementary Figure 3. Antidiabetic Therapy in Type II Diabetes Mellitus Patients (Excluding Insulin-Dependent Patients)**

**Supplementary Figure 4. Antidiabetic Therapy in Type II Diabetes Mellitus Patients (Including Insulin-Dependent Patients)**
